# 2D geometric shapes dataset – for machine learning and pattern recognition

**DOI:** 10.1016/j.dib.2020.106090

**Published:** 2020-07-25

**Authors:** Anas El Korchi, Youssef Ghanou

**Affiliations:** University Moulay Ismail of Meknes, Morocco

**Keywords:** Geometric shapes dataset, Machine learning, Pattern recognition, Classification synthetic dataset

## Abstract

In this paper, we present a data article that describes a dataset of nine 2D geometric shapes, and each shape is drawn randomly on a 200 × 200 RGB image. During the generation of this dataset, the perimeter and the position of each shape are selected randomly and independently for each image. The rotation angle of each shape is chosen randomly for each image within an interval between -180° and 180°, as well as the background colour of each image and the filling colour of each shape are selected randomly and independently.

The published dataset is composed of 9 classes of data, and each class represent a type of geometric shape (Triangle, Square, Pentagon, Hexagon, Heptagon, Octagon, Nonagon, Circle and Star). Each class is composed of 10k generated images. This paper also includes a GitHub URL to the generator source code used for the generation, which can be reused to generate any desired size of data.

The proposed dataset aims to provide a perfectly clean dataset, for classification as well as clustering purposes. The fact that this dataset is generated synthetically provides the ability to use it to study the behaviour of machine learning models independently of the nature of the dataset or the possible noise or data leak that can be found in any other datasets. Moreover, the choice of a 2D geometrical shape dataset provides the ability to understand as well to have good knowledge of the number of patterns stored inside each data class.

Specifications table

**Table:** 

**Subject**	Computer vision and pattern recognition
**Specific subject area**	Automatically generated 2D geometric shapes dataset that contains nine types of shapes (Triangles, Squares, Pentagons, Hexagons, Heptagons, Octagons, Nonagons, Circles and Stars)
**Type of data**	Image
**How data were acquired**	The geometric shapes were generated by using the turtle library and the Python programming language.
**Data format**	Raw
**Parameters for data collection**	Each geometric shape was drawn on a 200 × 200 pixel image, and each shape has a random background colour, random filling colour, a random position inside the image, an arbitrary rotation angle between -180° and 180°, and a random perimeter under the condition that the drawn shape must be fully visible inside the containing image.
**Description of data collection**	Each geometric shape was drawn 10 000 times under the conditions described above, and each image was saved into a PNG image.
**Data source location**	Institution: University Moulay Ismail of Meknes
	City: Meknes
	Country: Morocco
**Data accessibility**	Direct URL to data: https://data.mendeley.com/datasets/wzr2yv7r53/1
	Data generator source code: https://github.com/elkorchi/2DGeometricShapesGenerator

## Value of the data

• The simulated dataset can be used to train machine learning models to perform geometric shape recognition tasks.
• This dataset could be useful for researchers studying the overfitting of machine learning models [2] since the classification of the images inside this dataset can only be performed with a model that focuses on pattern detection [1] instead of memorization.
• This dataset could be useful for researchers to benchmark and test any regularization techniques that can be applied to machine learning models or any other approach that can help to avoid the overfitting.
• This dataset consists of a set of images containing random drawn geometrical shapes, which provides a clean dataset from any data leak that would influence the benchmark of any machine learning model.

## 1 Data description

**Fig. 1:**
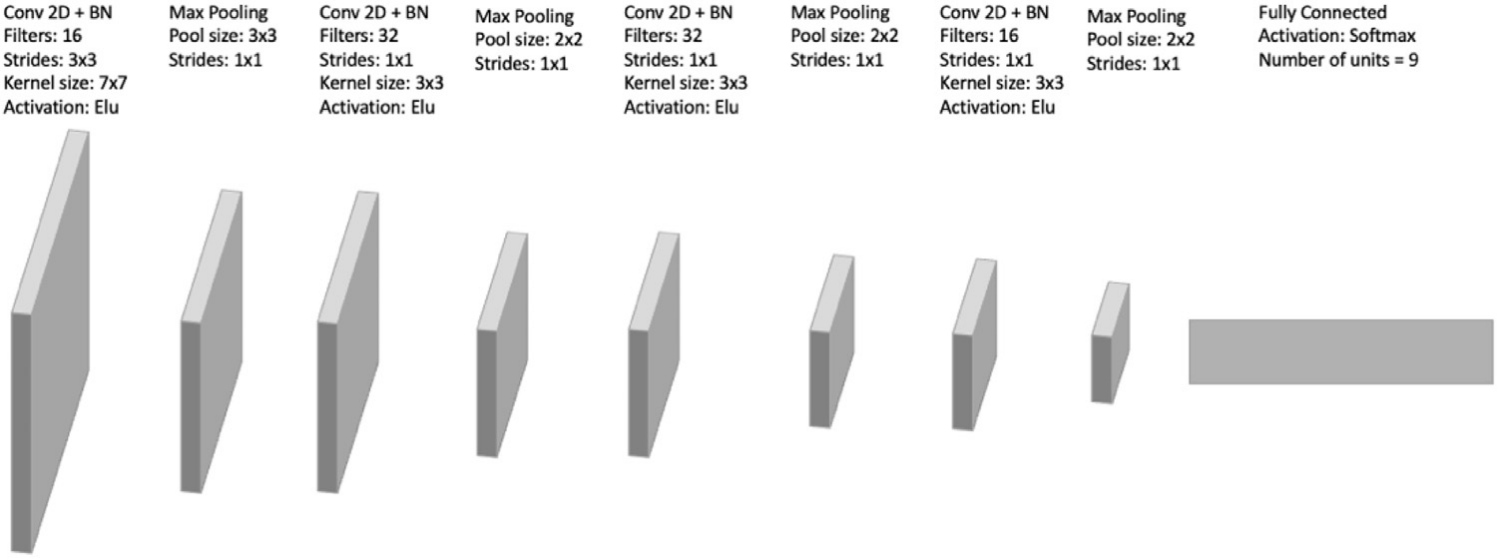
CNN architecture used for recognition and classification of geometric shapes. Conv 2D + BN denotes a convolutional layer with Batch Normalization [3].

The presented dataset is a set of 90k RGB images of a fixed resolution 200 × 200 pixel. This specific resolution aims to provide the minimum space where we can draw the geometric shapes with different positions and different sizes inside the image. Taking into consideration that high-resolution images require more computation power for machine learning models during the training phase, which may make the published dataset less useful as a benchmarking dataset. Each image contains one of the following polygon shapes: Triangles, Squares, Pentagons, Hexagons, Heptagons, Octagons, Nonagons, Circles and Stars. 10k images were generated for each shape (Fig. 1). Each image is saved in a PNG file. The name of each file is composed of two sections: [Type of polygon shape drawn inside the image]**_**[UUID].png.

Examples:

• Triangle_6f1d8142–2a58–11ea-8f1b-c869cd928de6.png
• Square_6f22e600–2a58–11ea-a950-c869cd928de6.png
• Pentagon_6f22e998–2a58–11ea-9fc2-c869cd928de6.png
• Hexagon_6f22eb64–2a58–11ea-818b-c869cd928de6.png
• Heptagon_6f22ecd8–2a58–11ea-82eb-c869cd928de6.png
• Octagon_6f22f118–2a58–11ea-944f-c869cd928de6.png
• Nonagon_6f22f318–2a58–11ea-bf89-c869cd928de6.png
• Circle_6f22f4d8–2a58–11ea-beb7-c869cd928de6.png
• Star_6f22f654–2a58–11ea-b6fc-c869cd928de6.png

The background colour of each image, the filling colour of each shape, the rotation angle of each shape, the position of each shape inside the image, as well the perimeter of each shape is generated randomly and independently.

## 2 Experimental design, materials, and methods

The generation program was developed using the Python programming language and the Turtle library to draw geometric shapes inside each image as well we have used the NumPy library to generate all the random parameters that we have used during the generation. The generation program receives as an input the number of desired samples per each shape as well as the path of the storage directory where the generated images will be stored. The goal of the tool is to generate a set of labelled images that contains 2D geometric shapes.

Each image is generated with the following parameters:

• A fixed image size of 200 × 200 pixel
• A random background colour: the background colour is generated randomly by using the function **numpy.random.randInt(0, 255, 3).** This function generates three random integers from the discrete uniform distribution in the half-open interval of 0 and 255. Each number represent respectively, the RGB values that construct the background colour. We ignore the possibility that the background colour may be different or close to the filling colour as we consider it as a normal task for a machine learning model, to be able to identify the drawn shape with a filling colour close to the background colour.
• A random filling colour of each shape: the filling colour of each shape is generated by using the same setting described in the generation of the background colour. Note that the generation of the filling colour is independently from the generation of the background colour (we ignore the very low probability that the background colour would be the same as the filling colour).
• A random rotation angle between -180° and 180°
• A random shape perimeter
• A random shape position inside the containing image

The perimeter and the position of each shape inside each image are selected as the following.

• First, we select the radius of the circumscribed circle of the drawn shape randomly, and this selection is performed within the interval of 10 and 75 (pixels), which ensures that the drawn shape may have a random perimeter between the interval of 10 and 75. Also, it gives that the smallest possible shape may be circumscribed by a circle with a radius = 10 pixel, and the largest possible shape may be circumscribed by a circle with a radius = 75 pixel. In Table 1 we show the minimal possible perimeter of each shape the maximal possible perimeter for each shape, the minimal possible area of each shape and the maximal possible area of each shape based on the parameters described above.

**Table 1:** Statistical description of the generated dataset.

Shape	Minimal perimeter (pixel)	Maximal perimeter (pixel)	Minimal area(pixel)	Maximal area (pixel)	Number of samples
Triangle	51.96	389.71	129.90	7 307	10 000
Square	56.57	424.26	200.00	11 250	10 000
Pentagon	58.78	440.84	237.76	13 374.23	10 000
Hexagon	60.00	450.00	259.80	14 614.18	10 000
Heptagon	60.75	455.58	272.00	15 300.06	10 000
Octagon	61.23	459.22	282.84	15 909.90	10 000
Nonagon	61.56	461.73	289.25	16 270.56	10 000
Circle	62.83	471.24	314.16	17 671.46	10 000
Star	126.29	947.18	112.26	6 314.46	10 000
• Then, in the function of the selected radius value, we choose the position of the centre of the circumscribed circle of the drawn shape inside the image randomly, the position is selected under the condition that all the drawn shape must be fully visible inside the image.
• And finally, we draw the shape in the function of the centre and position of its circumscribed circle. Note that the circumscribed circle of the shape is used only to draw the shapes, and it is not visible in the generated images (Fig. 2).

**Fig. 2:**
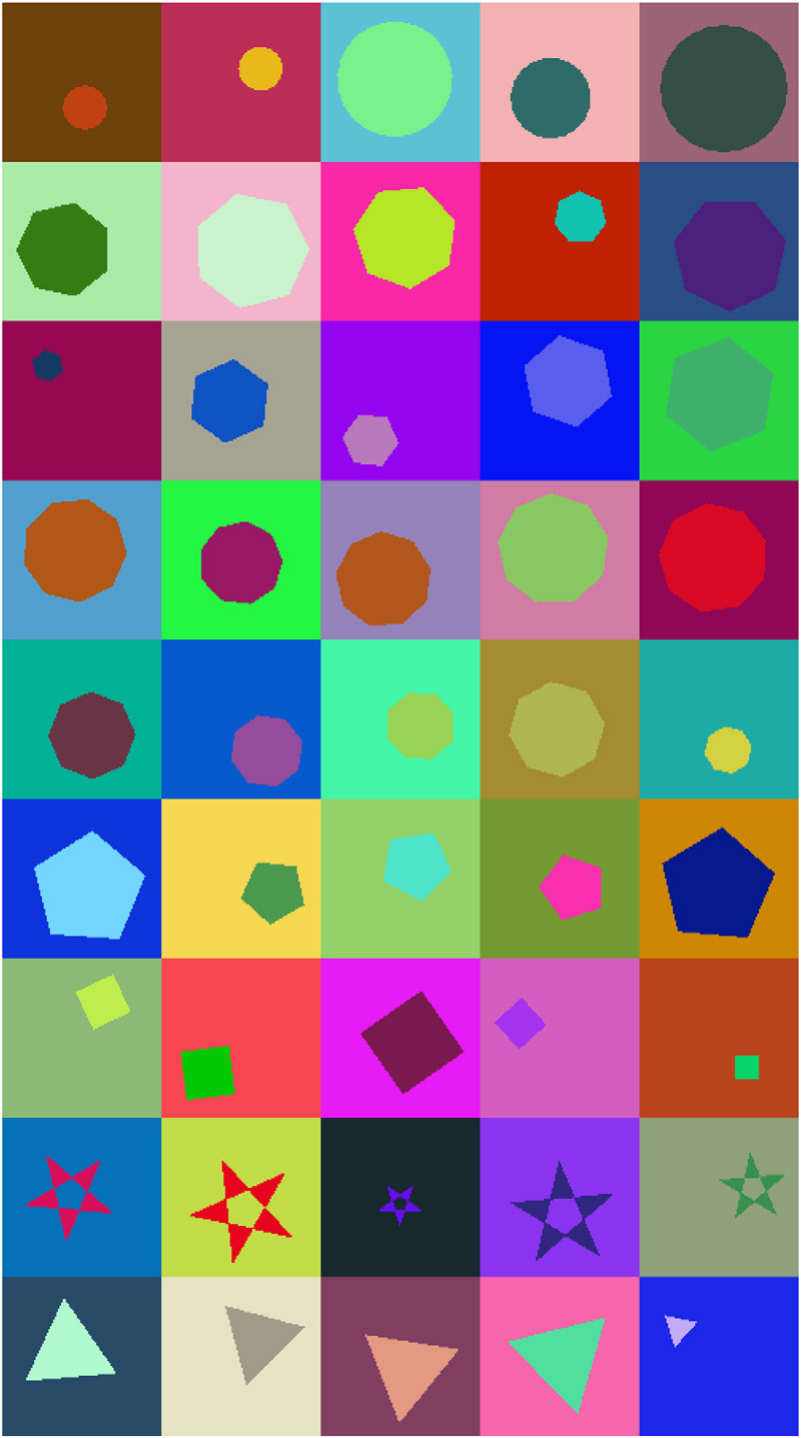
Samples from the generated dataset, each row contains examples that belong to the same geometric shape.

All the parameters described above are generated for each sample independently and identically. All the random values are selected by using the Numpy function ***numpy.random.randInt***, which generates a defined number of random values from the uniform discrete distribution in the half-open interval specified in the function call arguments.

## 3 A use case example with convolutional neural networks

This dataset could be used to train a convolutional neural network to perform geometric shape recognition and classification. To do so, this dataset must be divided into two separate sets of data a training dataset and a testing dataset, the selection of samples that composes each collection of data is made randomly:

• A training dataset which consists of 40% of samples of the original dataset
• A testing dataset which consists of 60% of samples of the original dataset

The architecture of the convolutional neural network is defined as the following: 7 convolutional layers and one fully connected layer, which represents the output layer of the neural network. Each convolutional layers is illustrated with a parametrized number of filters a batch normalization layer [3] an activation function, and a max-pooling layer follows each convolutional layer. Fig. 1 shows the convolutional neural network architecture that can be used on this dataset.

## Declaration of Competing Interest

The authors declare that they have no known competing financial interests or personal relationships which have, or could be perceived to have, influenced the work reported in this article.
